# Clinical Report on the First Prototype of a Photoacoustic Tomography System with Dual Illumination for Breast Cancer Imaging

**DOI:** 10.1371/journal.pone.0139113

**Published:** 2015-10-27

**Authors:** Elham Fakhrejahani, Masae Torii, Toshiyuki Kitai, Shotaro Kanao, Yasufumi Asao, Yohei Hashizume, Yoshiki Mikami, Iku Yamaga, Masako Kataoka, Tomoharu Sugie, Masahiro Takada, Hironori Haga, Kaori Togashi, Tsuyoshi Shiina, Masakazu Toi

**Affiliations:** 1 Department of Breast Surgery, Graduate School of Medicine, Kyoto University, Kyoto, Japan; 2 Department of Surgery, Kishiwada City Hospital, Osaka, Japan; 3 Department of Diagnostic Imaging and Nuclear Medicine, Graduate School of Medicine, Kyoto University, Kyoto, Japan; 4 Canon Inc., Tokyo, Japan; 5 Department of Diagnostic Pathology, Kumamoto University Hospital, Kumamoto, Japan; 6 Department of Surgery, Kansai Medical University, Osaka, Japan; 7 Department of Diagnostic Pathology, Graduate School of Medicine, Kyoto University, Kyoto, Japan; 8 Department of Human Health Sciences, Graduate School of Medicine, Kyoto University, Kyoto, Japan; Wayne State University, UNITED STATES

## Abstract

Photoacoustic tomography is a recently developed imaging modality that can provide high spatial-resolution images of hemoglobin distribution in tissues such as the breast. Because breast cancer is an angiogenesis-dependent type of malignancy, we evaluated the clinical acceptability of breast tissue images produced using our first prototype photoacoustic mammography (PAM) system in patients with known cancer. Post-excisionally, histological sections of the tumors were stained immunohistochemically (IHC) for CD31 (an endothelial marker) and carbonic anhydrase IX (CAIX) (a marker of hypoxia). Whole-slide scanning and image analyses were used to evaluate the tumor microvessel distribution pattern and to calculate the total vascular perimeter (TVP)/area for each lesion. In this clinical study, 42 lesions were primarily scanned using PAM preoperatively, three of which were reported to be benign and were excluded from statistical analysis. Images were produced for 29 out of 39 cancers (visibility rate = 74.4%) at the median depth of 26.5 (3.25–51.2) mm. Age, menopausal status, body mass index, history of neoadjuvant treatment, clinical stage and histological tumor angiogenesis markers did not seem to affect the visibility. The oxygen saturation level in all of the measured lesions was lower than in the subcutaneous counterpart vessels (Wilcoxon test, p value<0.001), as well as in the counterpart contralateral normal breast region of interest (ROI) (Wilcoxon test, p value = 0.001). Although the oxygen saturation level was not statistically significant between CAIX-positive vs. -negative cases, lesional TVP/area showed a positive correlation with the oxygen saturation level only in the group that had received therapy before PAM. In conclusion, the vascular and oxygenation data obtained by PAM have great potential for identifying functional features of breast tumors.

## Introduction

In 2012, breast cancer represented one out of every four cancer diagnoses worldwide [1]. Breast cancer is the second most common cancer in the world and the most frequent cancer among women but has a favorable survival rate when diagnosed in the early stages. Indeed, greater than 95% of breast cancer patients will survive for more than 5 years if diagnosed in the local stage [2]. However, highly sensitive screening methods have yet to be established. Conventional X-ray mammography (MMG), clinical breast exam (CBE) and self-breast exams, which are the accepted screening methods for early detection, have limited advantages in some types of breasts [3]. For instance, lesions in dense breasts are difficult to detect by MMG or even CBE [4], although ultrasonography (US) is a useful approach for evaluating dense breasts, as well as for confirming cysts. Other imaging modalities, such as magnetic resonance imaging (MRI), can be applied as an additional diagnostic step when an abnormality is detected by MMG or CBE. Although MRI can be a screening option for certain high-risk women, such as those with a positive family history of breast cancer or a history of therapeutic chest irradiation at earlier ages, it has not yet been proven to be beneficial for the general population [5].

In recent years, there has been a lot of progress in the development of optical imaging techniques for breast cancer diagnosis [6]. These techniques have drawn attention because they do not use ionizing radiation, like MMG, or need intravenous contrast agents, like MRI. Using near-infrared (NIR) light, it is possible to develop an image of the distribution of hemoglobin and oxygen saturation inside the breast tissue non-invasively, thus showing the potential for measuring angiogenesis in the breast [7]. The development of neovasculature has been shown to occur as early as when hyperplasia occurs in the mammary ducts [8]. Carpenter *et al*. showed that periductal microvessel density (MVD) was more than twenty times higher when compared to simple hyperplasia and normal ducts, which suggests that angiogenesis can be an ideal factor in the evaluation of breast abnormalities. Furthermore, there are reports indicating that MVD decreases significantly in patients who have responded to neoadjuvant chemotherapies [9, 10], thus making angiogenesis a potential imaging target to evaluate the grade of response to therapies.

Photoacoustic mammography (PAM) is a new technique in which a near-infrared laser is irradiated to the breast, and thermostatic waves produced by oxy (HbO_2_) and deoxy hemoglobin (Hb) inside the breast tissue are detected by ultrasound detectors [11]. As a result of angiogenesis, normal and abnormal breast tissues have different hemoglobin concentrations, and this provides a natural optical absorption contrast that may be imaged using PAM technology. Moreover, as ultrasound shows less scattering in the breast tissue, PAM shows higher spatial resolution than other optical techniques, such as diffuse optical tomography (DOT), in which light sources are used to obtain the hemoglobin distribution pattern in breast tissue [12].

We previously reported our initial clinical results of the first version of a prototype machine for a dual illuminated mode photoacoustic tomography system (Canon Inc., Tokyo, Japan; S1 Fig) used in Kyoto University Hospital [13]. Here, we report the final data from the first clinical trial that was conducted to study the usefulness of this machine in breast cancer patients in relation to the histological assessment of angiogenesis and hypoxia.

## Materials and Methods

### Patients

Patients with primary breast lesions who met the following criteria and provided written consent were enrolled in the study. The inclusion criteria were as follows: 20 years old or older at the time of diagnosis, a breast mass scheduled for surgical excision and Eastern Cooperative Oncology Group performance status of 0 or 1. The exclusion criteria included the following: scheduled for therapies other than surgery after PAM, pregnancy, suspicion of pregnancy, ongoing photodynamic therapy with photosensitizing agents such as Photofrin®, breast implants, cardiac pacemaker and any other conditions that made the patient unsuitable for participation in the study according to each clinical investigator. A total of 57 patients were considered for the study between August 2010 and March 2012 in Kyoto University Hospital, Japan. The primary surgery was performed within 1 month after PAM measurement. No patient received neoadjuvant therapy within this interval. All patients had undergone routine radiological (MMG, US and/or MRI) and histological diagnosis (core biopsy) prior to surgery. The study protocol was approved by the Medical Ethical Committee of Kyoto University (UMIN000003406).

### Pathologic diagnosis

After surgery, excised specimens were sectioned at 5 mm intervals perpendicular to the longest axis of a specimen, and permanent pathologic analysis was performed on formalin-fixed paraffin-embedded (FFPE) tissues by conventional hematoxylin-eosin (HE) staining. For invasive breast carcinoma (IBC) and ductal carcinoma *in situ* (DCIS), estrogen receptor (ER) and progesterone receptor (PgR) status were confirmed by immunohistochemistry (IHC). Human epidermal growth factor receptor 2 (HER2) status was confirmed by IHC or fluorescent *in situ* hybridization. Ki-67 immunostaining was performed using the MIB1 monoclonal antibody (Dako, Copenhagen, Denmark). for IBCs. The Ki-67 index was expressed as the percentage of Ki-67-positive malignant cells in 1,000 malignant cells assessed under high-power magnification (400×). A cut-off point of 14% was used to divide tumors into the low- vs. high-Ki67 group. Histological grade was calculated by a breast pathologist (13 years of experience) according to the General Rules for Clinical and Pathological Recording of Breast Cancer, 15^th^ edition, based on nuclear pleomorphic, tubule formation, and mitotic count scores for IBCs. The Histopathological Criteria for Assessment of Therapeutic Response of the Japanese Breast Cancer Society was applied to examine the pathological effect of primary systemic therapy (PST) for subjects who had received therapy before PAM.

### Histological assessment of angiogenesis

**Fig 1:**
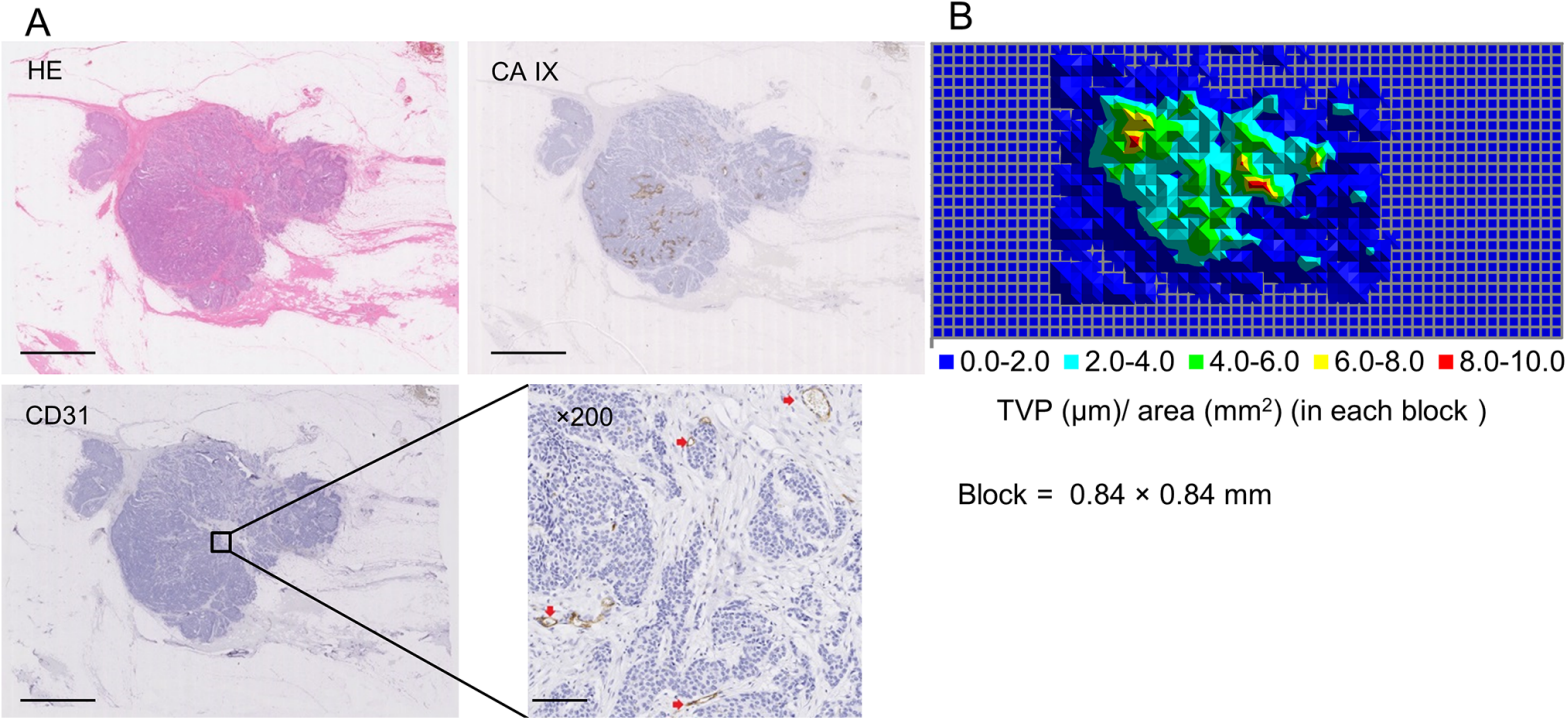
Histological assessment of angiogenesis. **(A)** A representative histological section with HE, CD31 and CAIX staining (scale bar = 5 mm). Magnified CD31-stained slide shows multiple microvessels (red arrowheads; 100x magnification; scale bar = 100 μm) **(B)** Map of total vessel perimeter of CD31-positive structures in each block (0.84 x 0.84 mm). Lesional TVP (μm) to lesional area (mm^2^) ratio was calculated by selecting the blocks that corresponded to the lesion area on the HE slide.

Two consecutive 4 μm sections from tumors containing FFPE blocks were received and mounted on glass slides for each case. Anti-CD31 monoclonal antibody JC70A (Dako) was used for visualizing microvessels by IHC for each tumor containing sections at a dilution of 1:50 overnight after heat-induced antigen retrieval in Dako Target Retrieval solution pH 9 for 30 minutes, as instructed by the manufacturer. Sections were immunohistochemically stained for carbonic anhydrase IX (CAIX), as a marker of hypoxia, with the monoclonal antibody M75 (BioScience, Bratislava, Slovakia) at a dilution of 1:150 for one hour at room temperature without any antigen retrieval. Cytoplasmic membrane staining in cancerous epithelial cells was considered positive (Fig 1A). Lesions were categorized into the positive vs. negative CAIX groups based on focal staining in any of the analyzed sections from a given lesion. Whole histological glass slides were mounted with a coverslip and scanned with Virtual Slide Scanner, NanoZoomer 2.0 (Hamamatsu Photonics, Hamamatsu, Japan) using a 20x objective lens. Histological regions of interest (ROIs) were highlighted on HE slides, and the area was measured using the NanoZoomer Viewer (Hamamatsu Photonics).

For CD31 whole-slide image analysis, 105 jpeg files with 24-bit RGB class and 8,000 x 8,000 pixel resolution were extracted from each virtual slide with the NanoZoomer Toolkit (Hamamatsu Photonics) and then converted to binary images with Image Pro-Plus 7.0 software (Media Cybernetics, Bethesda, MD, USA). The optimal hue, saturation and intensity (HSI) to detect the maximum CD31 stained structures were selected for each virtual slide. A macro was created for the automatic measurement of the perimeter of CD31-positive structures, regardless of shape, length or presence/absence of lumen, in all 105 jpegs. Each binary file was divided into 16 squares of 0.84x0.84 mm. Total vessel perimeter (TVP) in each block of 0.84x0.84 mm was calculated, and the data were automatically mapped for the whole histological slide using the macro. After comparison with the HE slide, the blocks that corresponded to the lesion were selected, and the whole lesional TVP was calculated. The ratio of lesional TVP (in μm) to lesional area (in mm^2^) was used as a marker for each lesion. A representative case is shown in Fig 1B and S2 Fig.

### PAM assessments

The technical specification of the first version of the prototype machine used for this study has been previously described in detail [13].In brief, a Ti:Sa laser optically pumped with a Q-switched Nd:YAG laser is used, having a tunable wavelength in the range of 700 to 900 nm. The measurable area was 30 mm by 46 mm for one scan, which took 45 seconds. Ultrasound detector has a rectangular shape and consists of 345 elements, with 15 in the horizontal direction and 23 in the vertical direction. Each element size is 2mm and central frequency is 1 MHz. For PAM imaging, the breast was placed between two holding plates craniocaudally with minimum compression after applying acoustic coupling gel while the patient was in prone position (Supplementary information, S1 Fig). Pulsed laser beams irradiated the breast from both sides, and photoacoustic signals were detected on the caudal side by an array transducer. The array number was 15 elements in the horizontal direction and 23 in the vertical direction. Each element size was 2 mm squared. The measurable area was 30 mm x 46 mm for one scan, which took 3 seconds for laser irradiation and 40 seconds for detector positioning. The array transducer could detect the signals up to the depth of 50 mm. In the first 16 cases, the scan was performed at three adjacent areas where the lesion was considered to be located in the tumor-bearing breast and one area in the normal contralateral breast. In later cases, the scanning system automatically moved the array transducer in an area of 120 mm x 46 mm, which took 90 seconds for one automatic scan. The voxel pitch was set to 0.25 mm in each direction.

After data acquisition, photoacoustic image reconstruction was carried out using a modified universal back projection algorithm [14]. Two-mm maximum intensity projection (MIP) images of PAM were constructed. Intensity, shape and continuity of the signals were the selection criteria for the classification of vessel- or microvessel-rich lesions. Linear, curling and branching shaped signals were defined as vessels, while focally scattered or grouped signals were attributed to microvessel-rich lesions. Each MIP stack was compared to the MRI images (obtained during radiological diagnosis), and the subcutaneous vessels that were visible both in the MRI and PAM were adjusted to locate the lesion [*i*.*e*., ROI] in the PAM image (Supplementary information S3 Fig). A breast radiologist (with more than 10 years of experience) determined whether PAM signals were associated with the lesion based on the morphology, intensity and distribution of the signals. The same signal depth was used to assign ROIs on the contralateral breast. Based on PAM technology, at the wavelengths of 756 and 797 nm, HbO2 and Hb are the main absorbents of the light, and optical fluence distribution in breast tissue is estimated based on absorption and scattering coefficients in each wavelength. The oxygen saturation of hemoglobin (SO_2_) of the lesions was calculated in assigned ROIs, as well as in the subcutaneous vessels, in both breasts using the equations below:

{μa(λ1)=εHbO(λ1)⋅CHbO+εHb(λ1)⋅CHbμa(λ2)=εHbO(λ2)⋅CHbO+εHb(λ2)⋅CHbsO2=CHbOCHbO+CHb

(*λ*_1_, *λ*_2_: 756 nm, 797 nm, *μ*_*a*_: absorption coefficient, *C*_*HbO*_, *C*_*Hb*_: concentration of HbO_2_ and Hb, *ε*_*HbO*_, *ε*_*Hb*_: molar extinction coefficients of HbO_2_ and Hb, respectively). After each laser was emitted to the breast tissue, the local absorption of the light was followed by local thermal excitation and expansion, which resulted in pressure rise, and finally, acoustic waves. The μ_a_ value for each laser was calculated from the following equation: P_0_ = Γμ_a_Φ, (P_0_: initial pressure rise; Γ: Gruneisen parameter = 0.20 at body temperature; Φ: light fluence). Then, μ_a_ was used in the abovementioned equations to calculate SO_2_.There was a 90-second time interval between the two data acquisitions after each laser emission. The variance of P_0,_ as well as Φ, can result in the variance of μ_a,_ which may explain why the calculated SO_2_ was more than 100% in a few cases.

### Statistical analysis

Data were analyzed using SPSS (Statistical Package for the Social Sciences) for Microsoft Windows, version 20.0 (SPSS, Chicago, IL, USA). The nonparametric Mann-Whitney *U*-test was used to compare variables between two groups, and the Kruskal-Wallis test was used when there were more than two groups. The Wilcoxon signed-rank test was used to determine the difference in SO_2_ between the lesional and subcutaneous vascular ROIs or between the lesional and contralateral breasts. The Pearson's chi-square test of association was used to determine if there was a relationship between two categorical variables. Pearson’s correlation analysis was used to determine a significant relationship between lesional TVP/area and calculated SO_2_ for continuous variables. Differences with a p value <5% were considered statistically significant. Values are expressed as median (range) unless otherwise specified.

## Results

A total of 57 women were eligible for this study, and 40 participated in the study. Seventeen cases were excluded from PAM measurement due to small breast size, anatomical location of the lesion, physical difficulty of the patient laying in the prone position or patient withdrawal. Two patients had masses in both breasts. In total, 42 mass-bearing breasts were scanned using PAM. Three lesions were found to be benign after pathological exam and were excluded from the statistical analysis. For 10 cases, no lesion-associated signal was detected after adjusting with the MRI image. Twenty-nine breasts showed acceptable lesion-associated PAM signals. Table 1 shows the basic patient characteristics in these two groups (*i*.*e*., lesion-associated signal present vs. absent). There were no differences between the breast area and breast tissue thickness of the tumor-bearing breasts in these two groups (S1 Table).

**Table 1:** Patient characteristics.

	Lesion-associated PAM signal present (n = 29)	Lesion-associated PAM signal absent (n = 10)	P value
Age	62 (36–83)	56 (43–78)	0.54
Menopausal status			0.55
*Pre-menopause*	6	3	
*Post-menopause*	23	7	
Body mass index	23.4 (17.7–31.6)	25.1 (17.8–27.3)	0.32
History of PST			0.44
*Yes*	9	4	
*No*	20	6	
Mass anatomical location			0.30
*Superior to nipple*	23	6	
*Inferior to nipple*	6	4	
Clinical stage			0.61^⌘^
*0*	8	2	
*I*	14	5	
*II*	7	3	
Lesional TVP/area	6.3 (2.4–15.9)	5.3 (2.1–18.4)	0.46

^⌘^Kruskal-Wallis test

Signals from ROIs were detected at a median depth of 26.02 mm (3.25–51.2) in cases with lesion-associated PAM signals. Thirty-three out of 39 lesions were confirmed to be IBCs. Tumor size, lymph node status, histological grade, ER, PgR and HER-2 status, Ki67-index, neoadjuvant treatment, CAIX expression and lesional TVP/area were not significantly different between IBCs in the two PAM image groups (S2 Table).

**Fig 2:**
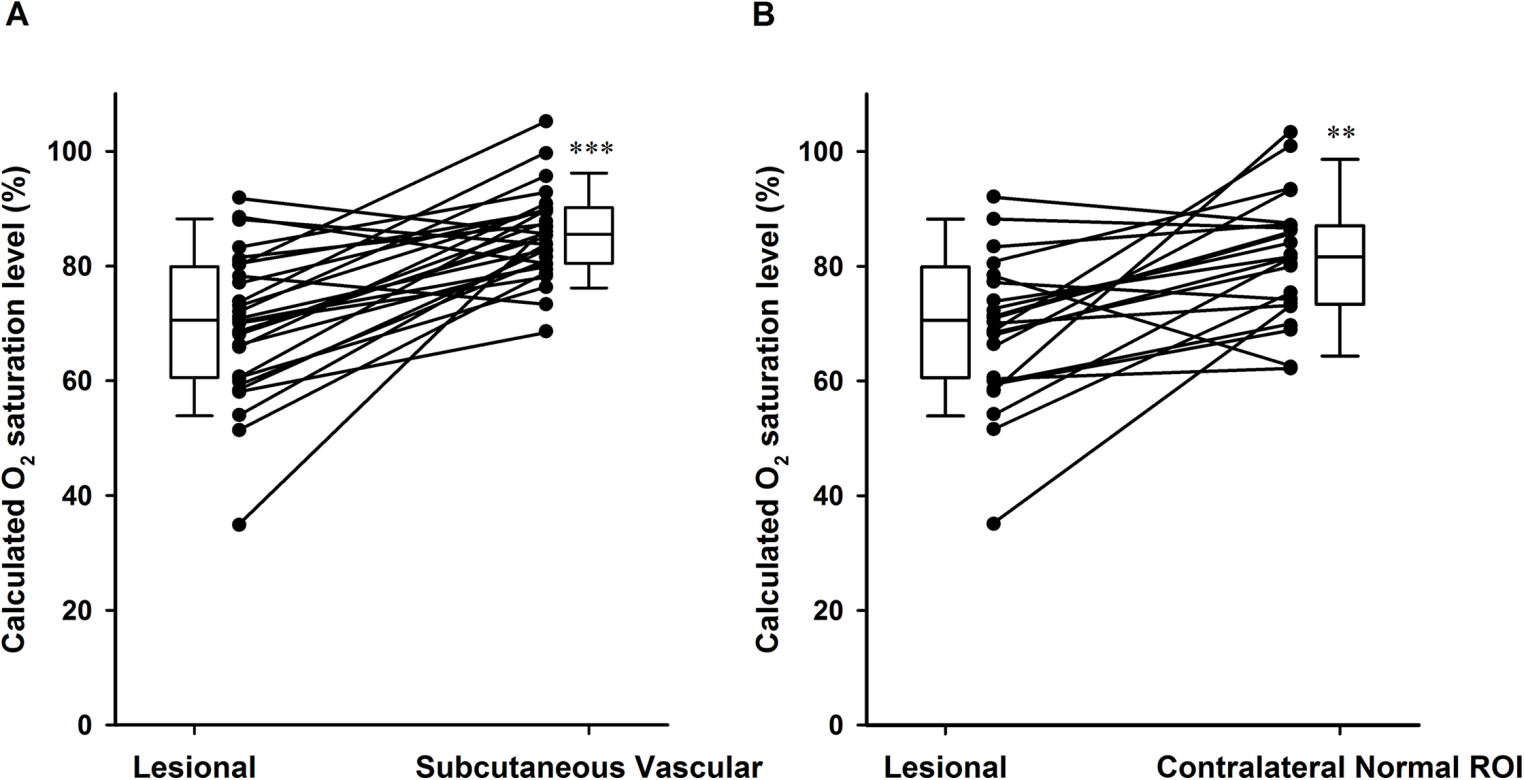
Oxygen saturation level of analyzed ROIs. **(A)** Oxygen saturation level in 28 lesions and the subcutaneous counterpart vessels. (Wilcoxon test, p value<0.001,❊❊❊). **(B)** Oxygen saturation level in 22 lesions and the counterpart contralateral normal breast ROIs (Wilcoxon test, p value = 0.001❊❊). Box plots present the median and 1^st^ and 3^rd^ quartiles.

The oxygen saturation level in all of the measured lesions was lower than that in the subcutaneous counterpart vessels (Wilcoxon test, p value<0.001, Fig 2A), as well as the counterpart contralateral normal breast ROIs (Wilcoxon test, p value = 0.001, Fig 2B). Normal breast ROIs showed the highest oxygen saturation level when compared with DCIS or IBC ROIs (S4 Fig). Table 2 summarizes the histological angiogenic profile of all cases. While none of the DCIS cases expressed CAIX, one third of IBCs in the study population expressed CAIX focally (11/33), with a significantly higher lesional TVP/area (Mann-Whitney *U*-test, p value = 0.029).

**Table 2:** Correlation between histological angiogenic profile in malignant cases (n = 39).

	DCIS (n = 6)	Lesional TVP/area	Invasive (n = 33)	Lesional TVP/area
CAIX expression				⌘
*Positive*	0		11	8.1 (3.4–18.4)
*Negative*	6	5.46 (2.1–15.9)	22	4.44 (2.4–13)

^⌘^ Mann-Whitney U-test, P value = 0.029

**Fig 3:**
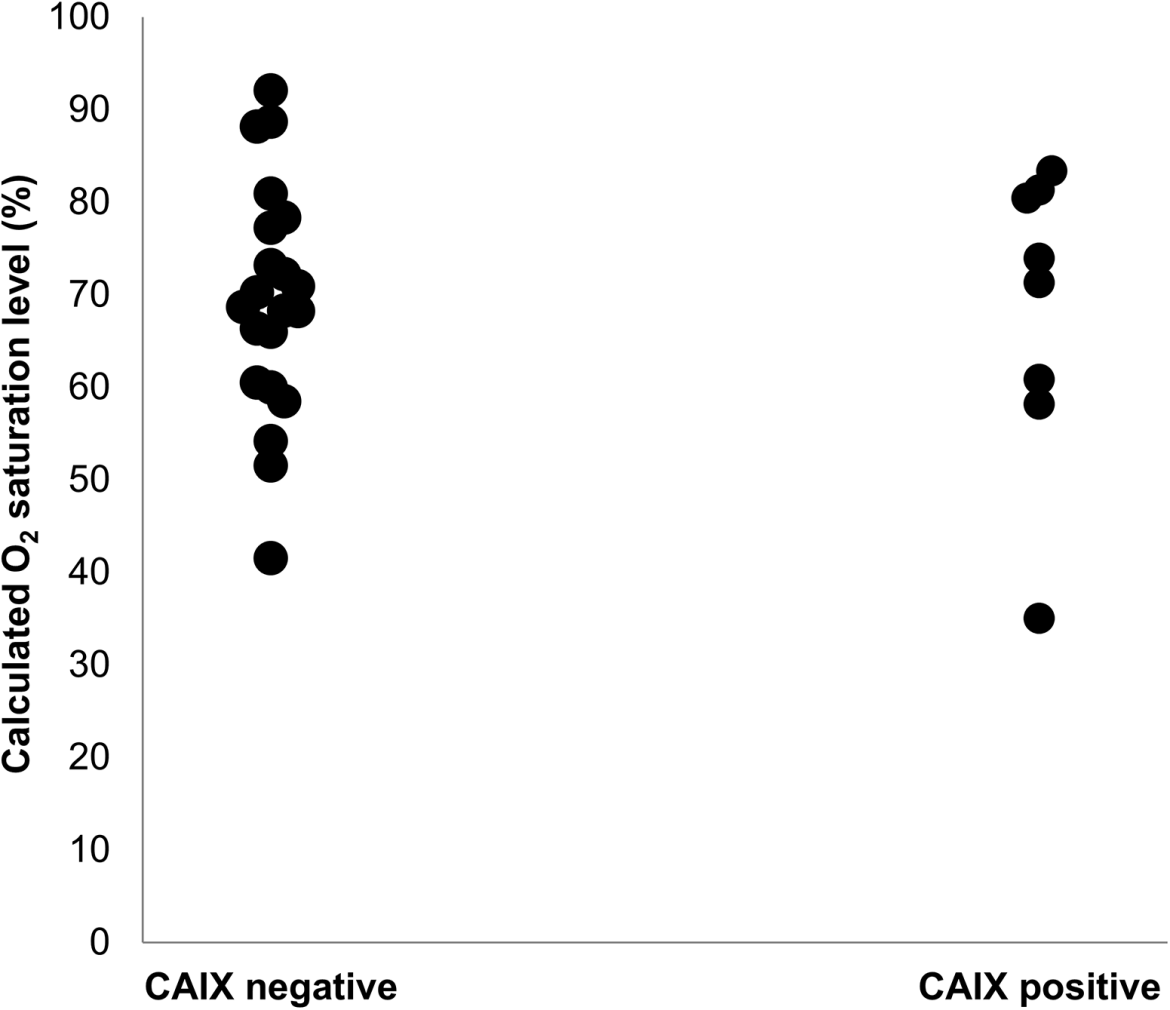
Oxygen saturation level in different CAIX expression groups. CAIX-negative group (n = 21): 68.67% (41.5%-92.05%); CAIX-positive group (n = 8); 72.61% (35.01%-83.36%). Mann-Whitney *U*-test, p value = 0.77

**Fig 4:**
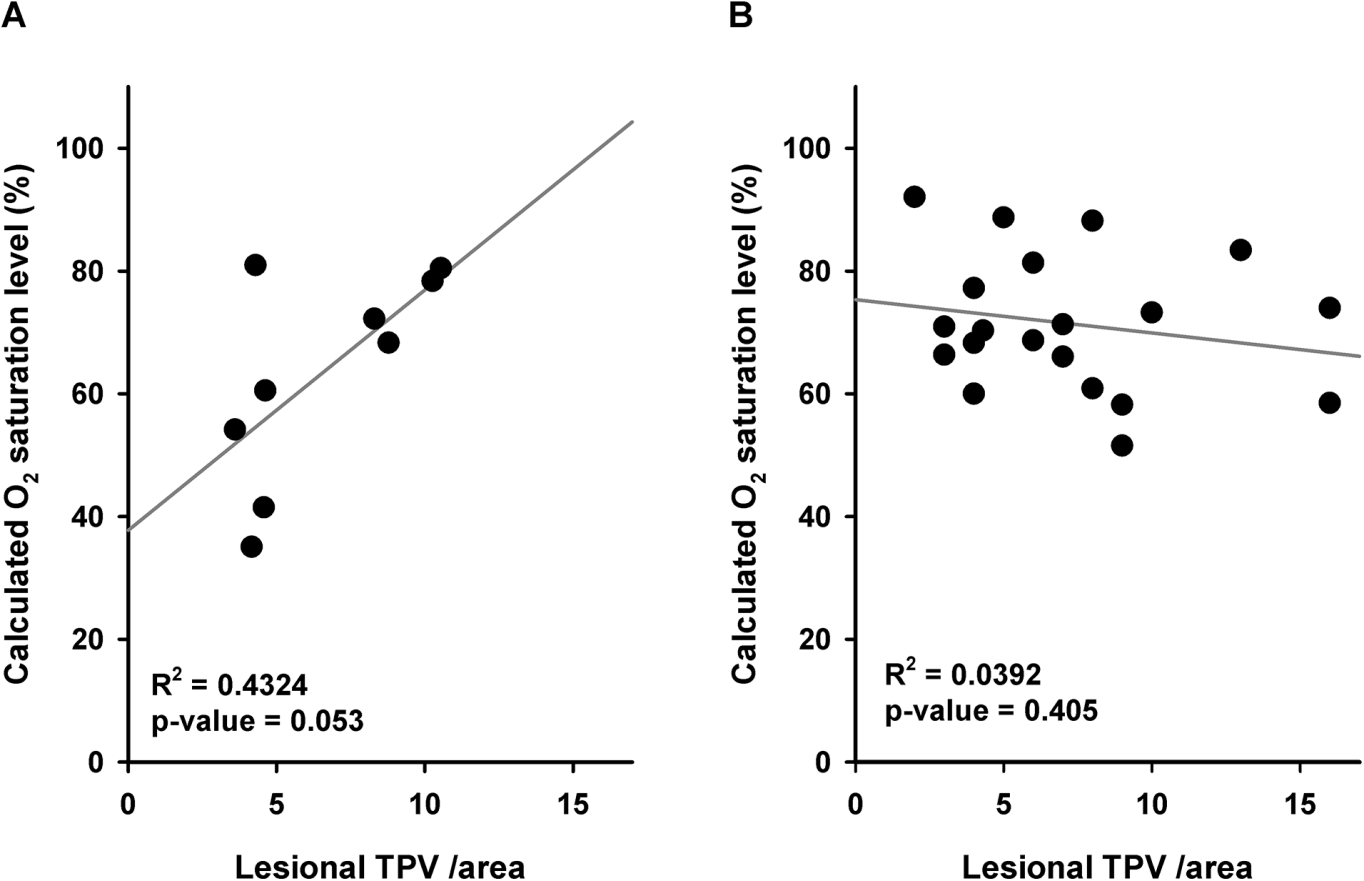
Correlation between lesional TVP /area and calculated oxygen saturation level. Pearson’s correlation coefficient between lesional TVP /area and calculated oxygen saturation level **(A)** in cases after PST (n = 9; R^2^ = 0.04234, p value = 0.053), and **(B)** in cases without PST (n = 20; R^2^ = 0.0392, p value = 0.405).

While 42.9% of the cases that had not received PST expressed CAIX, only 15.3% of the cases after PST were CAIX positive (S3 Table). The effect of PST on lesional TVP/area was consistent with CAIX expression (S4 Table). We performed the analysis in the divided groups based on PST history to test whether the histological angiogenic profile had any correlation with the calculated oxygen saturation. Although the oxygen saturation level was not significantly different between CAIX positive vs. negative cases (Fig 3), lesional TVP/area showed a positive correlation with oxygen saturation level only in the group that had received PST (Fig 4).

## Discussion

Angiogenesis, a hallmark of all cancers [15], occurs in the very early stages of breast cancer development [16]. In histological studies, periductal vascularity has been shown to be significantly higher when compared between normal and hyperplastic ducts in breast tissue from patients with DCIS [8,16]. Additionally, antiangiogenic therapies in which abnormal tumor vasculature is targeted for normalization are among the emerging therapeutic approaches for breast cancer [17]. Thus, imaging angiogenesis has been a topic of interest for the development of new techniques for detecting breast cancer, as well as for a noninvasive monitoring tool during treatment [18]. PAM is a new modality based on photoacoustic tomography [19] in which the breast is emitted with light at a wavelength that is best absorbed by the chromophores, such as Hb and HbO_2,_ which play a role in the optical contrast between malignant and normal breast tissue due to enhanced angiogenesis in cancerous lesions [20]. The absorbed light causes thermoelastic expansion, resulting in a pressure increase that can be detected by ultrasound detectors after propagation. High spatial resolution ultrasound in breast tissue enables the lesions to be quantitatively analyzed by PAM, and high spatial resolution of PAM is superior to other optical imaging modalities that use hemoglobin concentration differences between malignant and normal mammary tissue as the imaging contrast [21]. Several groups have designed and developed devices based on the photoacoustic technique and have tested the laboratory prototype in clinical settings [22,23], but to the best of our knowledge, this is the first clinical study to evaluate the clinical application of PAM to this extent.

In this initial clinical trial, the visual assessment of the breast and tumor vasculature, through mapping of PAM signals, was possible in 29 out of 39 breasts (visibility rate 74.4%). In the other 10 cases, detected signals did not show a specific pattern for classification as a microvessel-rich lesion. Theoretically, this difficulty must have arisen from the presence of the same-level signal from surrounding tissue in these cases. Technical reasons for this limitation could also be due to the following variables: incorrect operator setting, insufficient amount of applied gel or improper compression of the breast between the probe and detector. Breast size may also interfere with proper compression and may result in a higher background signal level. However, this study was performed in an Asian population, and body mass index was in the same range for both groups (Table 1). We also measured the breast area using the pictures taken with the built-in camera (S3 Fig). Neither breast area nor breast thickness (measured automatically after setting the breast between the holding plates) showed a difference between cases with vs. without lesion-associated signals (S1 Table). Non-technical factors could also be the result of an ill-structured breast vasculature. To detect mammary tissue-related factors, we analyzed both patient and lesion characteristics in the two groups. Although it was found by Doppler [24] that breast vessel variables are dependent on the menstrual status of cases studied, neither age nor menstrual status in our study seemed to be of statistical significance when compared between cases that were visible and non-visible by PAM.

Contrast-enhanced MRI studies that measure the number of total vessels longer than 3 cm in length with a maximum transverse diameter greater than 2 mm have shown that ipsilateral whole-breast vascularity asymmetrically increases in DCIS, as well as in IBC [25]. This finding may represent an underlying reason for an increased background signal level.

Diffuse optical spectroscopy imaging (DOSI) is another modality in which the breast is emitted with a wide range (600–1,100 nm) of light, and the optical signals are detected after passing through the tissue [26]. When measured with DOSI, the hemoglobin distribution pattern in tumor-bearing breasts was mapped as hot-spots or uniform based on the difference between the lesions and normal tissue [27]. In a clinical study of 118 breast cancer patients, 37.3% of the breasts showed a uniform pattern, which could imply a global rise in hemoglobin in these breasts [28]. These data are comparable to the 25.6% rate of non-visibility by PAM found in our study; the visibility rate by PAM (74.4%) was within the acceptable range in our study, given that the study population (initial 42 lesions) was a heterogeneous group of cases, some of which had received PST. Of note, one of the three benign lesions that were excluded from the statistical analysis was from a patient who showed a complete pathological response (pCR) after PST. In addition, 4 out of 10 non-visible cases had undergone neoadjuvant therapies, and the morphologic effects of PST on whole-breast stromal tissue may have been a factor in these cases [29]. Two of the non-visible cases were reported to be mucinous carcinoma, and colloid constituents of these tumors may have distorted the PAM signal [30]. However, the IBC visibility rate remained the same (72.7%), and no clinicopathological factor seemed to be of significant difference between the two groups. Only one out of six DCIS cases evaluated by PAM was non-visible, and this patient had achieved a grade 3 pathological response to PST.

The calculated SO_2_ values in the subcutaneous vasculature, both on the tumor side and contralateral breast side, were comparable (median 85.5% vs. 86.4%, respectively, data not shown). This result implies that breast compression during PAM did not affect blood circulation or oxygenation in the breast tissue. With this in mind, the lower lesional SO_2_ values in breast lesions compared to normal ROIs suggest reduced tissue oxygenation and hypoxia in malignant tumors. This result is in accordance with previously reported tissue oxygenation data from DOSI studies [28,31].

For testing the possible correlation between clinically measured lesional SO_2_ by PAM and histological angiogenic characteristics, we performed auto-image analysis on CD31-stained histological sections from all cases. Using this method, we were able to measure the histological quantitative characteristics of tumor microvessels (*i*.*e*., vessel count, vessel area, vessel perimeter) on the greatest dimension tumor section and the adjacent area, unlike manual measurement where hot spots are randomly selected by investigators. Our preliminary data showed that among the mentioned quantitative parameters, vascular perimeter was more coherent than the manually measured parameters in the selected areas (data not shown). We also calculated TVP and normalized this value to the surface area of each lesion (lesional TVP/area). Indeed, lesional TVP/area was shown to be correlated with CAIX expression, which is another accepted marker for hypoxia in breast cancer (Table 2) [32]. Neoadjuvant treatments decrease tumor cellularity, and in some cases, normalize the tumor microenvironment [33,34]. In 39 cases for which histological assessment was performed, the distribution pattern of lesional TVP/area, as well as CAIX, was different based on PST history (S3 and S4 Tables). However, a history of PST did not have an impact on the calculated lesional SO_2_ among the 29 cases (S5 Table).

A previous study demonstrated that the SO_2_ level was higher in 12 patients who achieved pCR after neoadjuvant therapy in comparison to 30 non-pCR cases [35]. In our study, none of the visible cases by PAM had achieved pCR, and due to the small sample size, we could not perform statistical analyses on the correlation between the grade of response to treatment and SO_2_ level. In this study, histological angiogenic parameters were not clearly related to calculated lesional SO_2_ (Fig 3, Fig 4), which may be due to the study population background (*e*.*g*., different PST regimens) or to nonhypoxic stimuli that influence CAIX expression [36]. In cases after PST, the positive correlation between lesional TVP/area and SO_2_ may be the reason for an increase in the number of functional microvessels; however, further studies on a more homogenous population, as well as an assessment of other microvessel characteristics such as shape [37] and maturity [38], are necessary to test this observation.

There were several potential limitations of this study. First, the breasts were mildly compressed in the craniocaudal direction between the two plates for PAM imaging, which differs from breast positioning in MRI, and we compared PAM signal with MRI images to locate the tumor in PAM after adjusting the subcutaneous vessels in both images. Although this was the best method available at the time, tumor location using both methods may not always be precisely identical. Another limitation was the imaging resolution, which was approximately 2 mm. The next-generation PAM machine should be designed with these limitations in mind.

In conclusion, vascular and oxygenation images obtained by PAM represent a new, non-invasive imaging method with great potential for providing functional features of breast tumors. Although technical improvements, such as higher resolution, are needed before testing its application for breast cancer diagnosis, oxygenation mapping of the whole breast could detect less-oxygenated areas in malignant cases. In the era of neoadjuvant therapies becoming a significant part of routine clinical practice, non-invasive tumor angiogenesis imaging modalities can provide helpful information for finding predictive, as well as prognostic, factors.

## Supporting Information

S1 Fig Schematic of the prototype machine used for this study. (JPG)

S2 Fig A representative case and histological assessment. (JPG)

S3 Fig Measurements of breast area and breast thickness. (JPG)

S4 Fig Oxygen saturation level in different groups ROIs. (JPG)

S1 Table Breast area and breast tissue thickness of tumor-bearing breasts in the study population. (DOCX)

S2 Table Histopathological characteristics of invasive carcinomas. (DOCX)

S3 Table CAIX expression in different PST groups. (DOCX)

S4 Table Lesional TVP/area in different PST groups. (DOCX)

S5 Table Lesional oxygen saturation level (SO_2_) in different PST groups. (DOCX)
