# Supplementary figures and images for: Clinical Report on the First Prototype of a Photoacoustic Tomography System with Dual Illumination for Breast Cancer Imaging
Source: PLoS One. 2015 Oct 27;10(10):e0139113. doi: 10.1371/journal.pone.0139113 (PMC4624636; doi:10.1371/journal.pone.0139113)

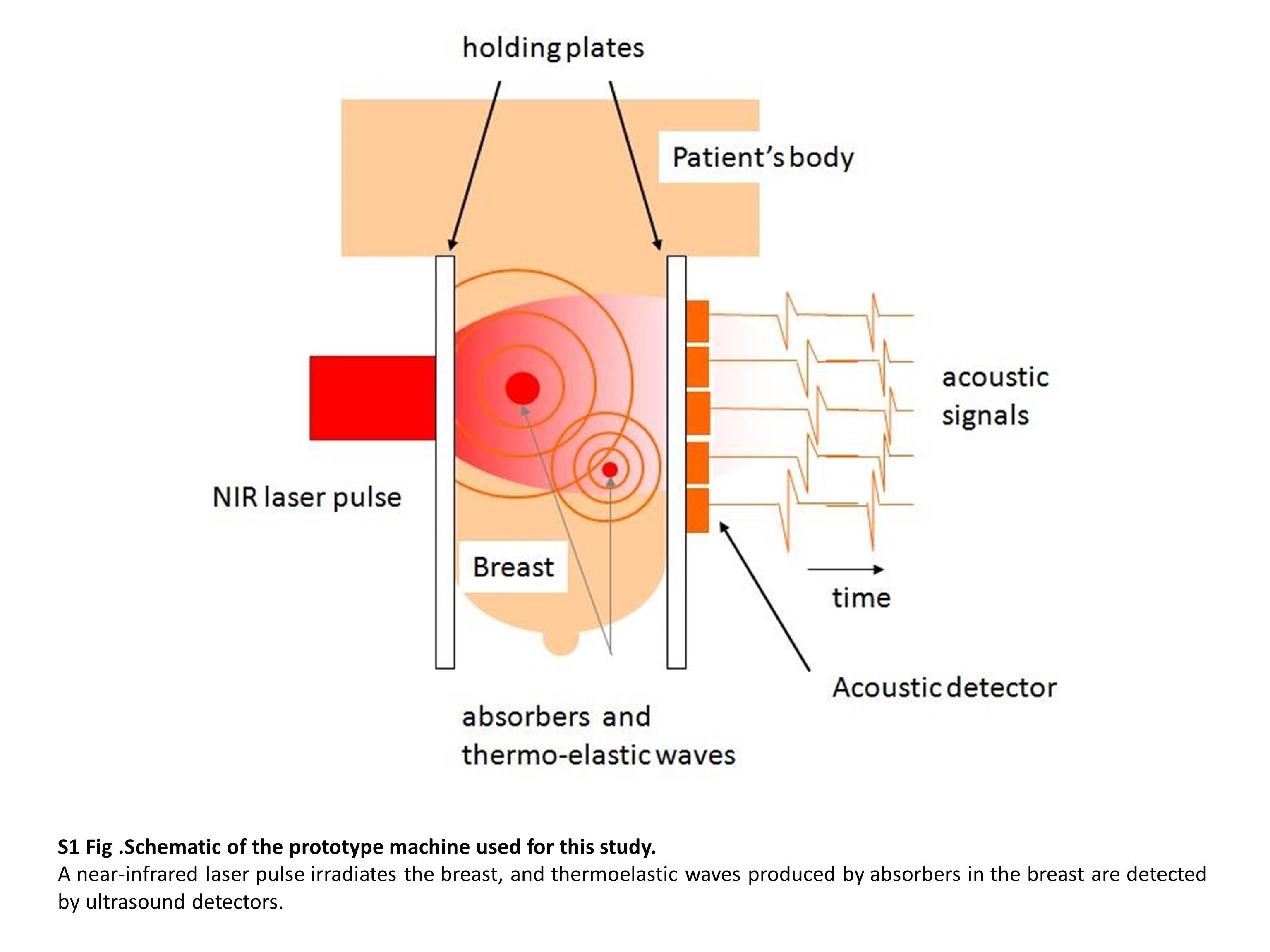

Supplement: S1 Fig — (JPG) [file pone.0139113.s001.jpg]

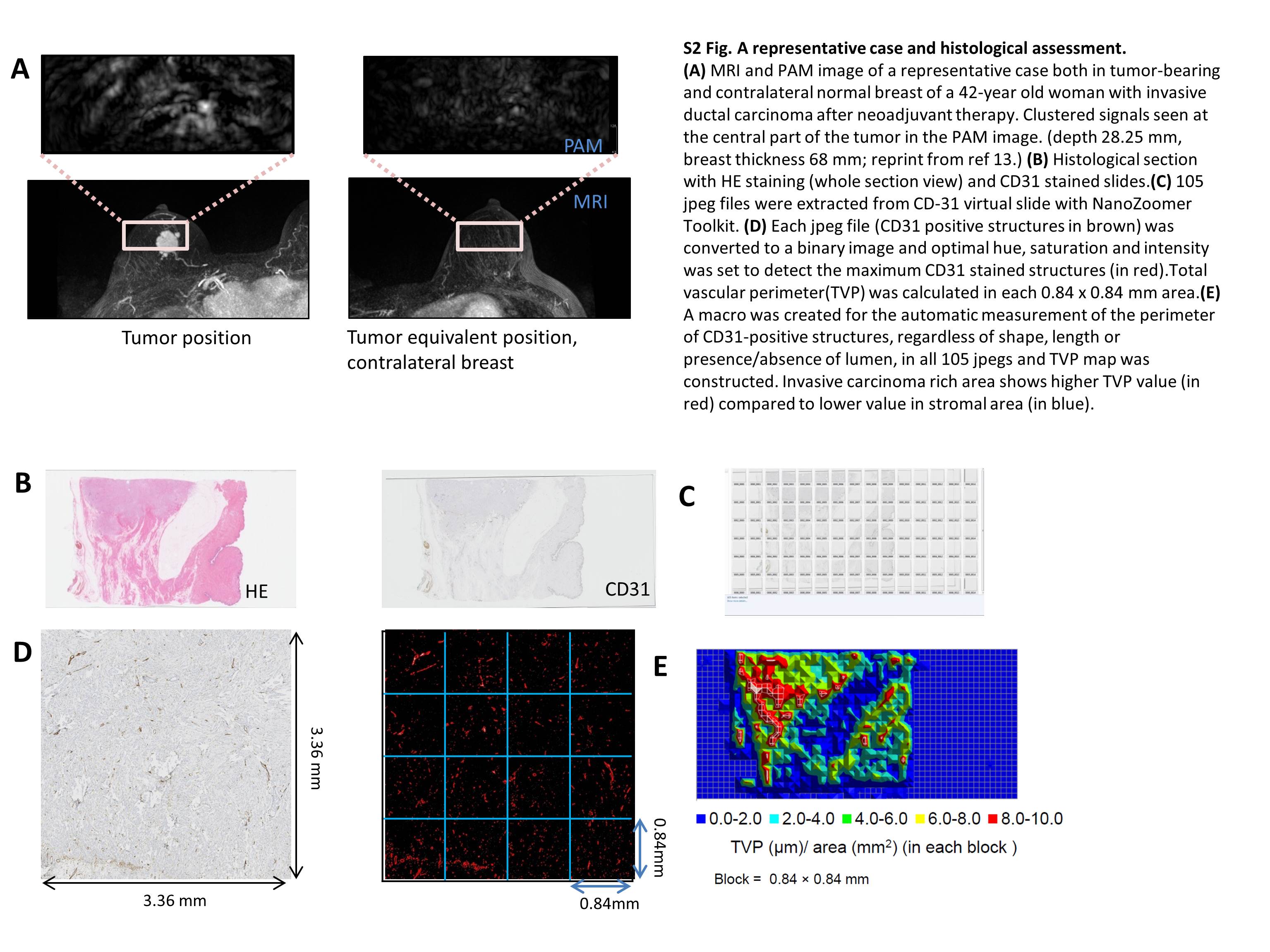

Supplement: S2 Fig — (JPG) [file pone.0139113.s002.jpg]

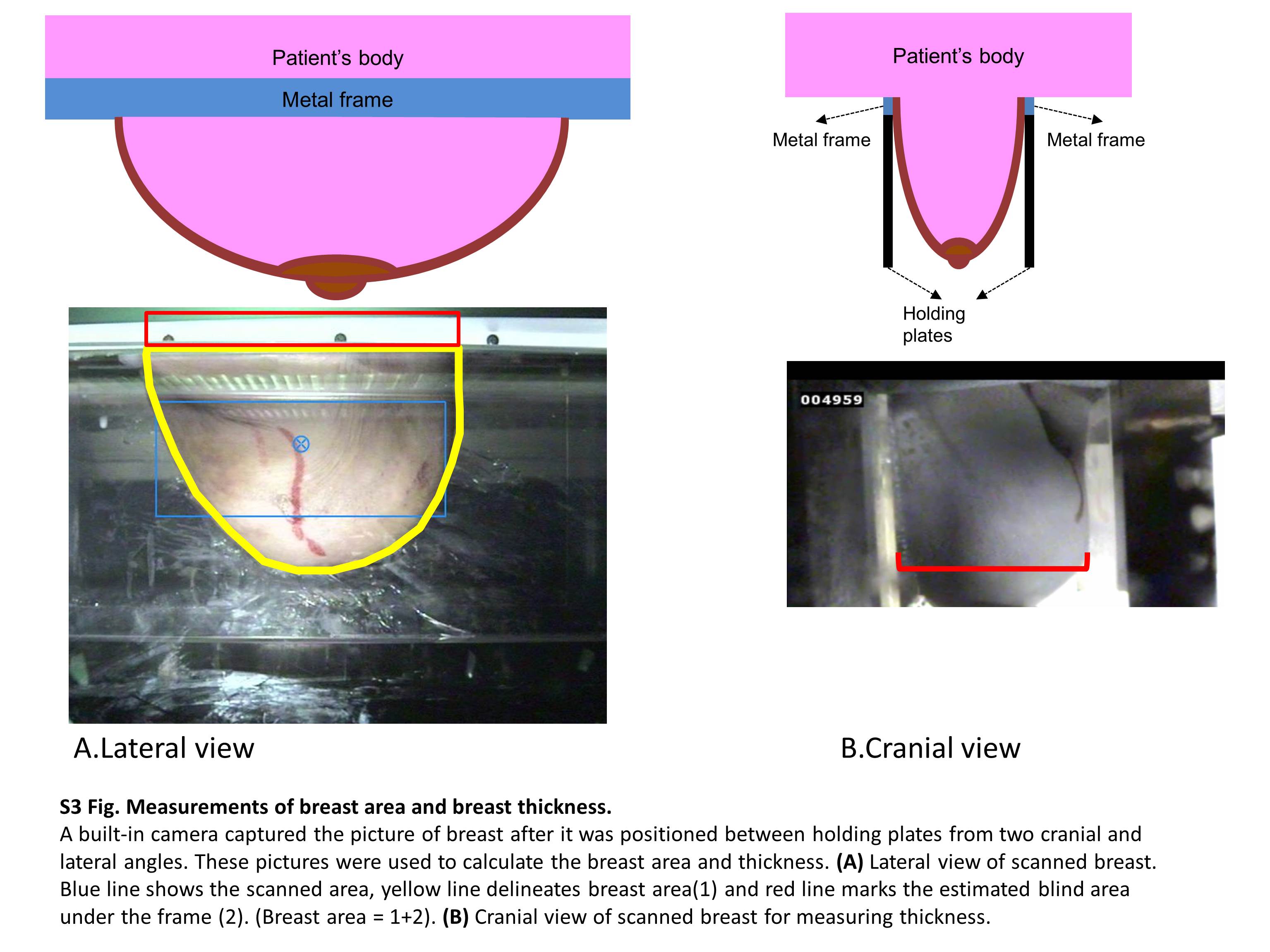

Supplement: S3 Fig — (JPG) [file pone.0139113.s003.jpg]

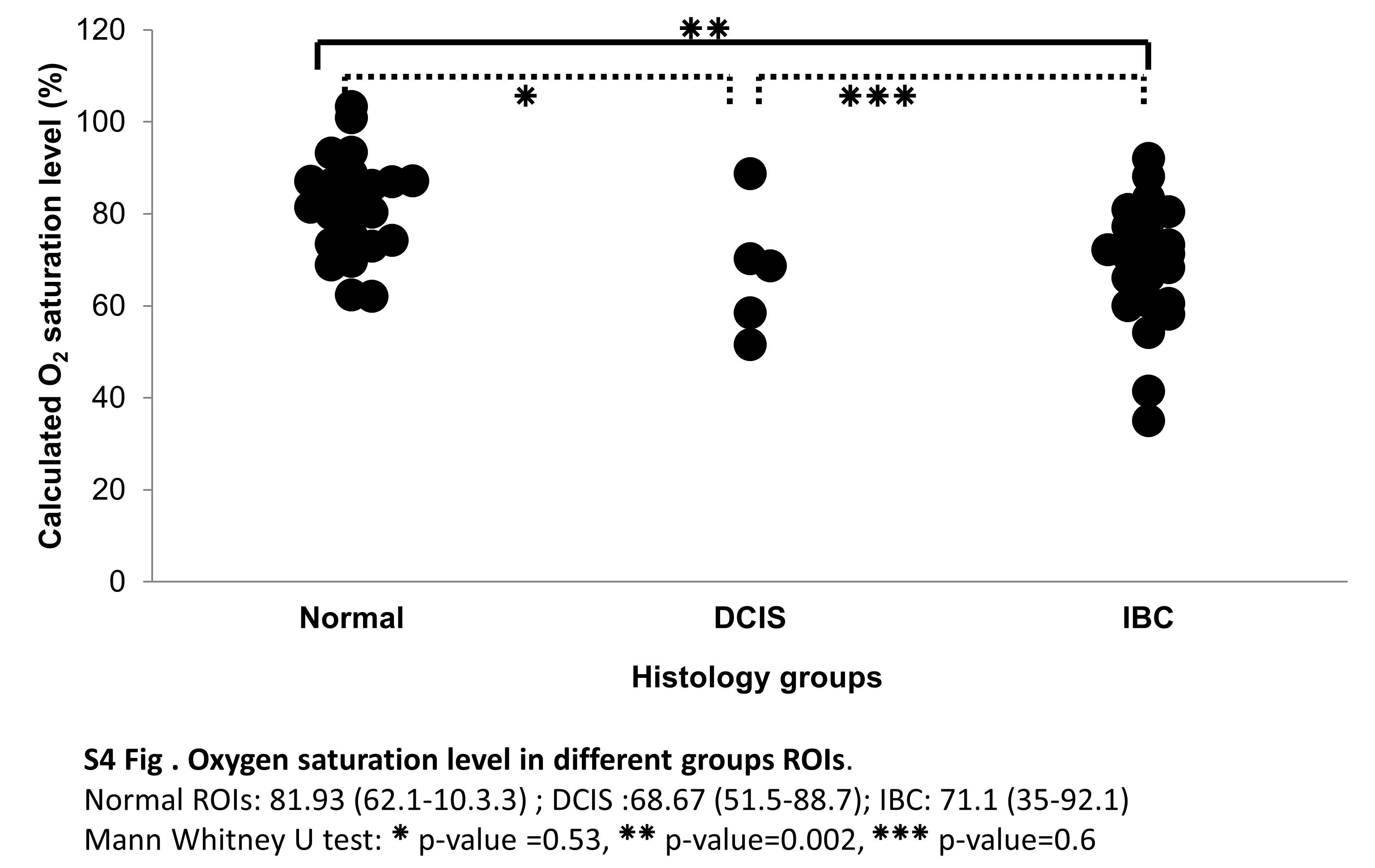

Supplement: S4 Fig — (JPG) [file pone.0139113.s004.jpg]
