# Supplementary material for: Clinical Report on the First Prototype of a Photoacoustic Tomography System with Dual Illumination for Breast Cancer Imaging
Source: PLoS One. 2015 Oct 27;10(10):e0139113. doi: 10.1371/journal.pone.0139113 (PMC4624636; doi:10.1371/journal.pone.0139113)
Supplement: S1 Table — (DOCX) [file pone.0139113.s005.docx]

**S1 Table. Breast area and breast tissue thickness of tumor-bearing breasts in the study population**

|  | **Lesion-associated PAM signal present (n=29)** | **Lesion-associated PAM signal absent (n=10)** | **P value***^⌘^* |
| --- | --- | --- | --- |
| Breast area (mm^2^) | 138 (82.7-238) | 137 (87.4-174) | 0.62 |
| Breast tissue thickness (mm) | 60.5 (26.9-79.4) | 65.2 (45.7-94.9) | 0.91 |

Median (Min-Max),^⌘^ Mann Whitney *U*-test
