# Supplementary material for: Clinical Report on the First Prototype of a Photoacoustic Tomography System with Dual Illumination for Breast Cancer Imaging
Source: PLoS One. 2015 Oct 27;10(10):e0139113. doi: 10.1371/journal.pone.0139113 (PMC4624636; doi:10.1371/journal.pone.0139113)
Supplement: S2 Table — (DOCX) [file pone.0139113.s006.docx]

**S2 Table. Histopathological characteristics of invasive carcinomas**

|  | **Lesion-associated PAM signal present (n=24)** | **Lesion-associated PAM signal absent (n=9)** | **P value** |
| --- | --- | --- | --- |
| Tumor size  <2  ≥2 | 17  7 | 4  5 | 0.16 |
| Histological grade  1  2  3 | 6  13  5 | 3  5  1 | 0.45^⌘^ |
| Lymph node metastasis  Positive  Negative | 8  16 | 3  6 | 0.65 |
| ER  Positive  Negative | 21  3 | 8  1 | 0.70 |
| PgR  Positive  Negative | 16  8 | 7  2 | 0.43 |
| HER2  Positive  Negative | 3  21 | 1  8 | 0.58 |
| Ki-67 index  <14%  ≥14% | 14  10 | 4  5 | 0.37 |
| Neoadjuvant therapy  Positive  Negative | 9  15 | 3  6 | 0.58 |
| CAIX expression  Positive  Negative | 8  16 | 3  6 | 0.66 |
| Lesional TVP / area | 6.44 (2.4-15.8) | 6.39 (3.2-18.4) | 0.86 |

^⌘^Kruskal –Wallis Test
