# Supplementary material for: Clinical Report on the First Prototype of a Photoacoustic Tomography System with Dual Illumination for Breast Cancer Imaging
Source: PLoS One. 2015 Oct 27;10(10):e0139113. doi: 10.1371/journal.pone.0139113 (PMC4624636; doi:10.1371/journal.pone.0139113)
Supplement: S3 Table — (DOCX) [file pone.0139113.s007.docx]

**S3 Table. CAIX expression in different PST groups**

|  | **Received PST**  (n=13) | **No treatment**  (n=26) | **P value** |
| --- | --- | --- | --- |
| CAIX positive | 2 (15.3%) | 9 (34.6%) | 0.19*^⌘^* |
| CAIX negative | 11 (84.7%) | 17 (65.4%) |  |

^⌘^Pearson's Chi-Square test
