# Supplementary material for: Clinical Report on the First Prototype of a Photoacoustic Tomography System with Dual Illumination for Breast Cancer Imaging
Source: PLoS One. 2015 Oct 27;10(10):e0139113. doi: 10.1371/journal.pone.0139113 (PMC4624636; doi:10.1371/journal.pone.0139113)
Supplement: S4 Table — (DOCX) [file pone.0139113.s008.docx]

**S4 Table. Lesional TVP/area in different PST groups**

|  | **Received PST**  (n=13) | **No treatment**  (n=26) | **P value** |
| --- | --- | --- | --- |
| Lesional TVP/area | 4.63 (2.14-13.05) | 6.51 (2.37-19.99) | 0.73*^⌘^* |

^⌘^ Mann Whitney *U*-test
