# Supplementary material for: Clinical Report on the First Prototype of a Photoacoustic Tomography System with Dual Illumination for Breast Cancer Imaging
Source: PLoS One. 2015 Oct 27;10(10):e0139113. doi: 10.1371/journal.pone.0139113 (PMC4624636; doi:10.1371/journal.pone.0139113)
Supplement: S5 Table — (DOCX) [file pone.0139113.s009.docx]

**S5 Table. Lesional oxygen saturation level (SO_2_) in different PST groups**

|  | **Received PST**  (n=8) | **No treatment**  (n=21) | **P value** |
| --- | --- | --- | --- |
| Lesional SO_2_ | 70% (41%-81%) | 70%(35%-92%) | 0.75*^⌘^* |

^⌘^ Mann Whitney *U*-test
